# The influence of climate and habitat on stable isotope signatures and the isotopic niche of nestling White‐headed Woodpeckers (*Dryobates albolarvatus*)

**DOI:** 10.1002/ece3.6624

**Published:** 2020-08-24

**Authors:** Teresa J. Lorenz, Jeffrey M. Kozma, Patrick G. Cunningham

**Affiliations:** ^1^ U.S. Department of Agriculture, Forest Service Pacific Northwest Research Station Olympia WA USA; ^2^ Yakama Nation, Timber, Fish and Wildlife/Fisheries Resource Management Toppenish WA USA; ^3^ U.S. Department of Agriculture, Forest Service Pacific Northwest Research Station Corvallis OR USA

**Keywords:** climate affects, isotopic niche, stable isotopes, White‐headed Woodpecker

## Abstract

The majority of landbird species feed their nestlings arthropods and variation in arthropod populations can impact reproductive outcomes in these species. Arthropod populations in turn are influenced by climate because temperature affects survival and reproduction, and larval development. Thus, climate factors have the potential to influence many bird species during their reproductive phases. In this study, we assessed climate factors that impact the diet of nestling White‐headed Woodpecker (*Dryobates albolarvatus*), an at‐risk keystone species in much of its range in western North America. To do this, we measured stable isotope signatures (δ^13^C and δ^15^N) in 152 nestlings across six years and linked variation in isotopic values to winter (December–February) and spring (June) precipitation and temperature using mixed effects models. We also explored habitat factors that may impact δ^13^C and δ^15^N and the relationship between δ^15^N and nest productivity. Last, we estimated isotopic niche width for nestlings in different watersheds and years using Bayesian standard ellipses, which allowed us to compare dietary niche width and overlap. We found that colder winter temperatures were associated with an increase in δ^15^N and δ^15^N levels had a weak positive relationship with nest productivity. We also found that sites with a more diverse tree community were associated with a broader isotopic niche width in nestlings. Our findings suggest that nestling diet is affected by climate, and under future warming climate scenarios, White‐headed Woodpecker nestling diet may shift in favor of lower trophic level prey (prey with lower δ^15^N levels). The impact of such changes on woodpecker populations merits further study.

## Graphical abstract

We studied the impact of habitat and climate on stable isotope signatures of nestling White‐headed Woodpeckers in the northwestern U.S.A. We found that colder winter temperatures were associated with higher trophic level diet in nestling White‐headed Woodpeckers.

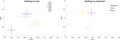

## 1 INTRODUCTION

As a result of growing greenhouse gas concentrations created by human activities, mean annual global temperature is expected to increase between 1.8 and 4.0°C during the 21st century (Bentz et al., 2010). Along with an increase in temperature, changes in precipitation patterns may result in a greater frequency and duration of droughts in the western United States (Seager et al., 2007). Insect populations are directly influenced by climate because temperature determines rate of larval development, as well as survival and reproduction of adults (Brown, Gillooly, Allen, Savage, & West, 2004; Chuine & Régnière, 2017). Because many birds, especially woodpeckers, respond positively to outbreaks of insects (Edworthy, Drever, & Martin, 2011; Morris, Cheshire, Miller, & Mott, 1958; Norris, Drever, & Martin, 2013; Saab, Latif, Dresser, & Dudley, 2019), changes in climate may alter the diet of these species.

While there are many methods for studying avian diet, stable isotope analyses (SIA) are a powerful approach that can be used to explore questions that are difficult to answer with traditional studies. Stable isotopes of nitrogen (^15^N/^14^N) and carbon (^13^C/^12^C) have been used to study avian diets since the late 1970s (Kelly, 2000). The stable isotopes of nitrogen are most often used to determine trophic feeding position (Hodum & Hobson, 2000; Mariano‐Jelicich, Botto, Martinetto, Iribarne, & Favero, 2008; St. John Glew et al., 2019). The stable isotopes of carbon have been used to determine the contributions of C_3_ and C_4_ plants to an animal's diet (Teeri & Schoeller, 1979), compare contributions of marine and terrestrial food sources (reviewed in Kelly, 2000), and determine general habitat conditions used for foraging (e.g., mesic vs. arid environments, or open vs. closed forest habitats; Chamberlain, Bensch, Feng, Akesson, & Andersson, 2000, Pagani‐Núñez, Barnett, & Senar, 2019). Both of these isotopes are also extremely useful in comparing foraging niche width and overlap among groups of animals (Jackson, Parnell, Inger, & Bearhop, 2011) and in documenting long‐term changes in diet (English, Green, & Noccera, 2018; Norris, Arcese, & Preikshot, 2007).

In this study, we used stable isotopes of nitrogen and carbon to explore the influence of climate and habitat factors on the diet of nestling White‐headed Woodpeckers (*Dryobates albolarvatus*). The White‐headed Woodpecker is an ecosystem engineer and keystone species that excavates cavities in trees and snags, which are important nest and roost structures for a large guild of secondary cavity users (Kozma, 2014; Tarbill, Manley, & White, 2015). In the northern portion of its range in western North America, the White‐headed Woodpecker is a sensitive, at‐risk species that inhabits a restricted set of forest types; predominately ponderosa pine (*Pinus ponderosa*) forests and to a lesser degree in mixed‐conifer forests where ponderosa pine and Douglas‐fir (*Pseudotsuga menziesii*) are codominant (Kozma & Kroll, 2012; Lorenz, Vierling, Kozma, Millard, & Raphael, 2015). Within the northwestern U.S. and southwestern Canada where White‐headed Woodpeckers are an at‐risk species, temperatures are expected to warm by 3.0°C in the next 60 years (Mote & Salathé, 2010). Therefore, an understanding of how climate and habitat factors influence White‐headed Woodpecker diet and niche width (i.e., the range of food items consumed) can help inform management of this species. This is especially true for the reproductive phase in the White‐headed Woodpecker's annual cycle. This species has limited reproductive capabilities compared to many forest songbirds because they produce just one brood per year, with an average of 2–3 nestlings produced per nest in northern locales (Kozma & Kroll, 2012; Lorenz, Vierling, Kozma, & Millard, 2016), and past studies suggest they are nest site limited due to low levels of standing dead trees (snags), even in otherwise suitable habitat (Lorenz et al., 2015).

Overall, an understanding of the factors that impact nestling diet and productivity in White‐headed Woodpecker may be of considerable importance in ensuring the persistence of this species in the face of climate change. Yet, only one study to date, Kozma and Kroll (2013), has explored White‐headed Woodpecker nestling diet. They found that the invertebrates most frequently fed to nestlings were wood‐boring beetle larvae (24.7%; Cerambycidae and Buprestidae), caterpillars (23.1%) and, ants and their larvae (18.2%), but were unable to examine climate or habitat factors associated with variation in nestling diets. Thus, information is lacking on spatial (habitat), temporal (seasonal), and climate factors that impact nestling diet, and the extent to which nestling diet varies among years or locations. Information is also lacking on whether differences in nestling diet affect nest productivity. To fill some of these information gaps, we designed a study to (1) model habitat and climate factors that may impact nestling feather δ^15^N and δ^13^C values, (2) assess whether variation in δ^15^N (indicative of trophic position) affects nest productivity (number of nestlings fledged per nest), and (3) compare isotopic niches of nestling White‐headed Woodpeckers across watersheds and years.

## 2 METHODS

### 2.1 Study area

**Figure 1:**
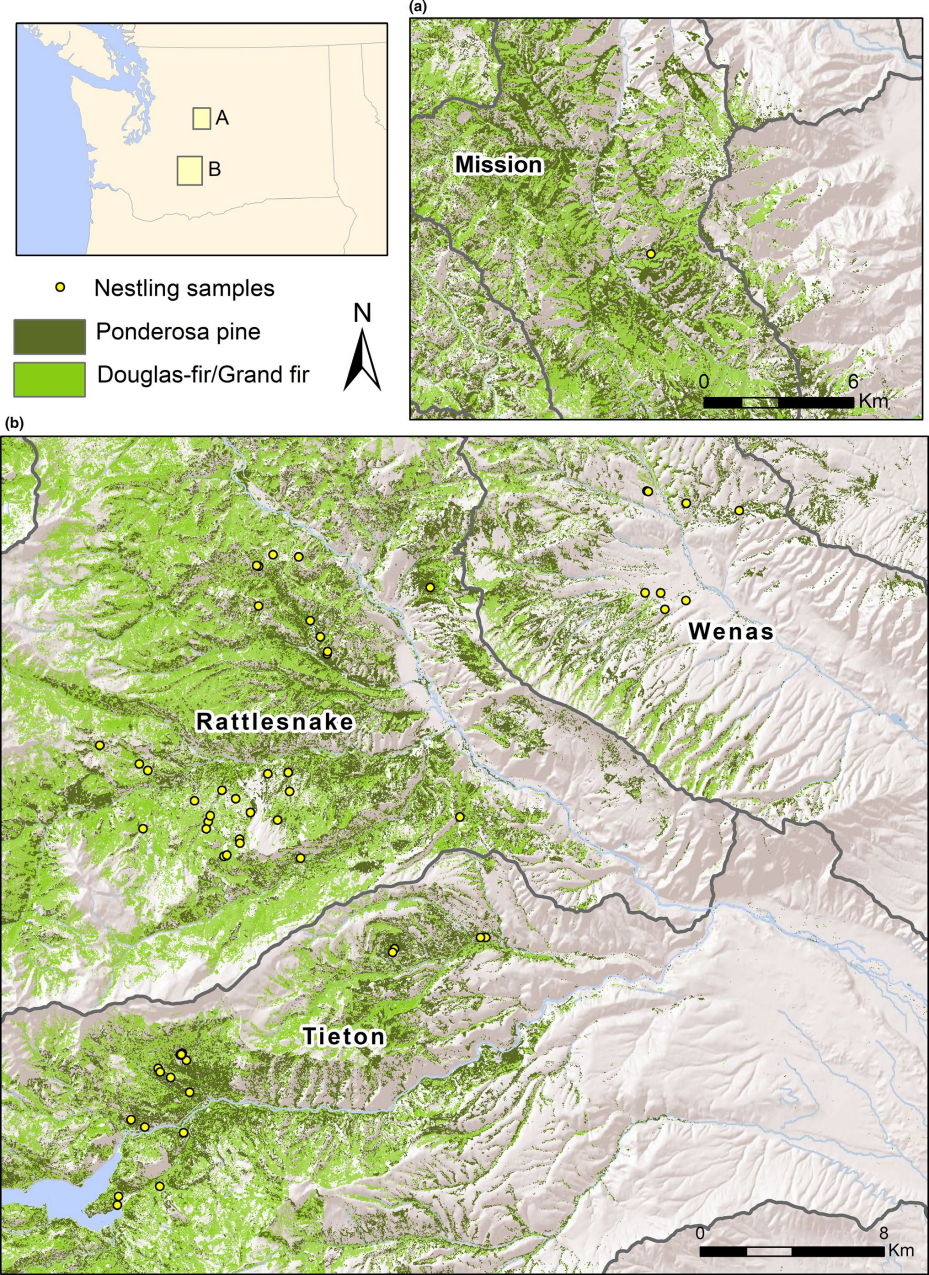
Location of four watersheds used to examine White‐headed Woodpecker stable isotope signatures in central Washington, from 2011 to 2017. The polygons show the outline of each watershed, and yellow dots are samples from 152 nestling woodpeckers

We conducted this study from 2011 to 2017 along the east slope of the Cascade Range in Yakima and Chelan counties, Washington, USA (~46°45′N, 120°58′W and 47°30′N, 120°33′W). In this region, over 80% of the precipitation falls during winter, with summers characterized as hot and dry (Wright & Agee, 2004). We searched for White‐headed Woodpeckers in areas where the species was known to occur from past research or in which reconnaissance surveys revealed breeding woodpeckers. Within our study area (Figure 1), we established study site boundaries for statistical analysis using hydrologic unit codes (HUC; Seaber, Kapinos, & Knapp, 1987). HUCs are a means of dividing the Unites States into successively smaller hydrologic units or watersheds. We used HUC 10, or 5th field watersheds, to define our study site boundaries, which resulted in four study sites (hereafter referred to as watersheds; Figure 1): Tieton, Rattlesnake, Wenas, and Mission. We included watershed as a random effect in our analysis (see below) to neutralize potential deviations from independence among nests within watersheds. The majority of our study area was administered by the U.S. Department of Agriculture Forest Service (USDAFS), with smaller portions being managed by Washington Department of Natural Resources (WDNR), Washington Department of Fish and Wildlife, and private landowners.

Forest composition varied within each watershed based on aspect, slope, elevation, and longitudinal distance east of the Cascade Crest, which provides a rain shadow for eastern Washington. Watersheds to the west were closer to the Cascade Crest, higher in elevation, and received more moisture than those farther east. For most watersheds, ponderosa pine was the dominant tree species. In areas receiving more rainfall, ponderosa pine was codominant with Douglas‐fir (*Pseudotsuga menziesii*) or grand fir (*Abies grandis*; hereafter, “firs”). Less common tree species included western larch (*Larix occidentalis*), quaking aspen (*Populus tremuloides*), and black cottonwood (*Populus trichocarpa*).

Most of our study area was actively managed for timber production. We estimated that ≥92% of our study area had been harvested for timber at least once since 1950 based on USDAFS timber harvest activity reports and WDNR forest practice applications. Most harvests were described as overstory removal cuts (removal of all mature trees) or partial removal cuts (partial removal of the overstory). Age of the dominant forest layer was estimated at <100 years (Lorenz et al., 2015), and portions of some watersheds had been burned with mixed‐severity prescribed fire, wildfire, and/or thinned by harvest within 10 years of the start of this study. Portions of some watersheds were actively grazed by domestic cattle or sheep during summer.

### 2.2 Data collection

We captured White‐headed Woodpecker nestlings at nest sites in June and July, from 2011 to 2012 and 2014 to 2017 (6 yr). We captured nestlings using the hole saw method (Ibarzabal & Tremblay, 2006), except for a small number that we captured by hand (*n* = 6) or mist net (*n* = 1) during fledging. For each nestling, we pulled 3–5 contour feathers from the flank and we recorded the number of nestlings within each nest. All capture and handling methods were approved by the University of Idaho Animal Care and Use Committee (Permit Number 2011‐30), U.S. Department of Agriculture Animal Care and Use Committee (Permit number 2016‐007), U.S. Geological Survey Bird Banding Lab (Permit numbers 22104 and 24061), and Washington State Department of Fish and Wildlife.

### 2.3 Stable isotope analyses

We stored feathers in breathable paper envelopes until SIA was performed during winter 2018/2019. Prior to SIA, samples were cleaned of surface oils in a 2:1 chloroform:menthol solution. We submitted samples to the Cornell University Stable Isotope Laboratory (COIL), which uses a Thermo Delta V isotope ratio mass spectrometer interfaced to a NC2500 elemental analyzer. In‐house standards were routinely calibrated against reference materials provided by the International Atomic Energy Association. COIL estimated accuracy and precision using an in‐house deer standard analyzed after every 10 samples resulting in an overall standard deviation for our runs of 0.11% for δ^15^N and 0.19% for δ^13^C. They used a chemical methionine standard to quantify instrument accuracy across a gradient of amplitude intensities. Delta values obtained between the amplitudes of 150 and 15,000 mV for δ^15^N had an error of 0.33%, and delta values between 100 and 15,000 mV had an error of 0.34% for δ^13^C. COIL performed isotopic corrections using a two‐point normalization (linear regression), using KCRN (a ground corn standard) and CBT (Cayuga brown trout) as in‐house standards with known δ^13^C and δ^15^N values determined using global standards (PeeDee Belemnite for δ^13^C and atmospheric nitrogen for δ^15^N). We report all stable isotope values in the δ notation and in parts per thousand according to the equation: δ^13^C or δ^15^N = ([*R*_sample_/*R*_standard_] − 1)·1,000, where *R* is 13C/12C or 15N/14N.

### 2.4 Habitat data

To assess the impact of habitat on nestling δ^13^C or δ^15^N, we obtained remotely sensed data on forest attributes around each woodpecker nest location using forest attribute data from LEMMA (2012: https://lemma.forestry.oregonstate.edu/). These datasets were generated from gradient nearest neighbor (GNN) structure maps (Ohmann, Gregory, Henderson, & Roberts, 2011), derived from a combination of field plots, mapped environmental data, and Landsat Thematic Mapper satellite imagery. These data provided spatially explicit information on vegetation features at a 30‐m resolution for all watersheds in our study. To account for spatial uncertainty regarding woodpecker foraging locations (we only had location of nests sites), we estimated the mean for each of our habitat variables (Table 1) in a 125‐ha area (37 pixel area) centered around each nest, the average breeding home range size for White‐headed Woodpeckers in our study area (Lorenz et al., 2015).

**Table 1:** Description of covariates considered for modeling factors influencing feather δ^13^C and δ^15^N ratios in nestling White‐headed Woodpeckers in central Washington, 2011–2017

Covariate	Description	Included in nestling δ^15^N model?	Included in nestling δ^13^C model?
*Habitat factors*			
Abgr_psme_ba (m^2^/ha)	Basal area of grand fir and Douglas‐fir, averaged in 150 ha area around nest site	Yes	Yes
Nest_elevation (m)	Elevation of the nest site from which the bird was captured	Yes	Yes
Pipo_ba (m^2^/ha)	Basal area of ponderosa pine, averaged in 150 ha area around nest site	Yes	Yes
Qmdc_dom (cm)	Quadratic mean diameter of all dominant and codominant conifers, averaged in 150 ha area around nest site	Yes	Yes
*Climate factors*			
June_meant (C)	Mean temperature in June	Yes	No
June_ppt (mm)	Mean precipitation in June	No	Yes
Winter_meant (C)	Mean temperature for the months December–February for the winter prior to the bird's capture (mean temperature of the mean monthly temperature)	Yes	Yes
Winter_ppt (mm)	For the months December–February in the winter prior to the bird's capture, sum of precipitation	Yes	Yes

As with any large‐scale modeling effort, the GNN datasets had some inherent inaccuracy. This inaccuracy was quantified in accuracy assessments that estimated correlation coefficients, normalized root mean squared errors, and coefficients of determination (LEMMA, 2012). Some of the GNN covariates that we used had relatively low accuracy and therefore may not reflect on‐the‐ground habitat attributes in our study area. We therefore considered a separate class of models using elevation (obtained from a digital elevation model) as a proxy to reflect habitat at nests. While nest elevation is a fairly crude means of ascribing habitat conditions to nests, for our purposes it had some advantages over GNN, such as being spatially accurate while being correlated with precipitation and longitude within the range of ponderosa pine habitat used by White‐headed Woodpeckers. We therefore developed a set of models (see below) using GNN data and a separate set of models using elevation in place of GNN data. We then used Akaike's information criterion adjusted for small sample sizes (AIC_c_) to rank support for such models (Burnham & Anderson, 2002), as described below in our statistical analysis.

### 2.5 Climate data

We used data from PRISM (PRISM Climate Group, 2019: https://prism.oregonstate.edu/) to model climate factors associated with variation in δ^15^N and δ^13^C. We obtained spatially explicit data on mean temperature and precipitation from PRISM for the nests in our study and for all years. PRISM data were available in 2.5 arcmin (4 km) resolution for our study area. Feathers collected from nestlings represent diet during the nestling's developmental period, which occurs mostly in June. With this in mind, we obtained PRISM data on June mean temperature and precipitation. We also obtained PRISM data on the average winter temperature and precipitation (December–February) because weather in the winter prior to our feather collection may affect diet (as arthropod survival can be affected by winter temperature and snowpack; Flower, Gavin, Heyerdahl, Parsons, & Cohn, 2014; Kolb et al., 2016; Régnière & Bentz, 2007). While temperature extremes may have a stronger bearing on arthropod populations in some situations (e.g., prolonged extreme winter cold may kill overwintering arthropods), average temperatures were highly correlated with minimum and maximum temperatures in this data set.

**Figure 2:**
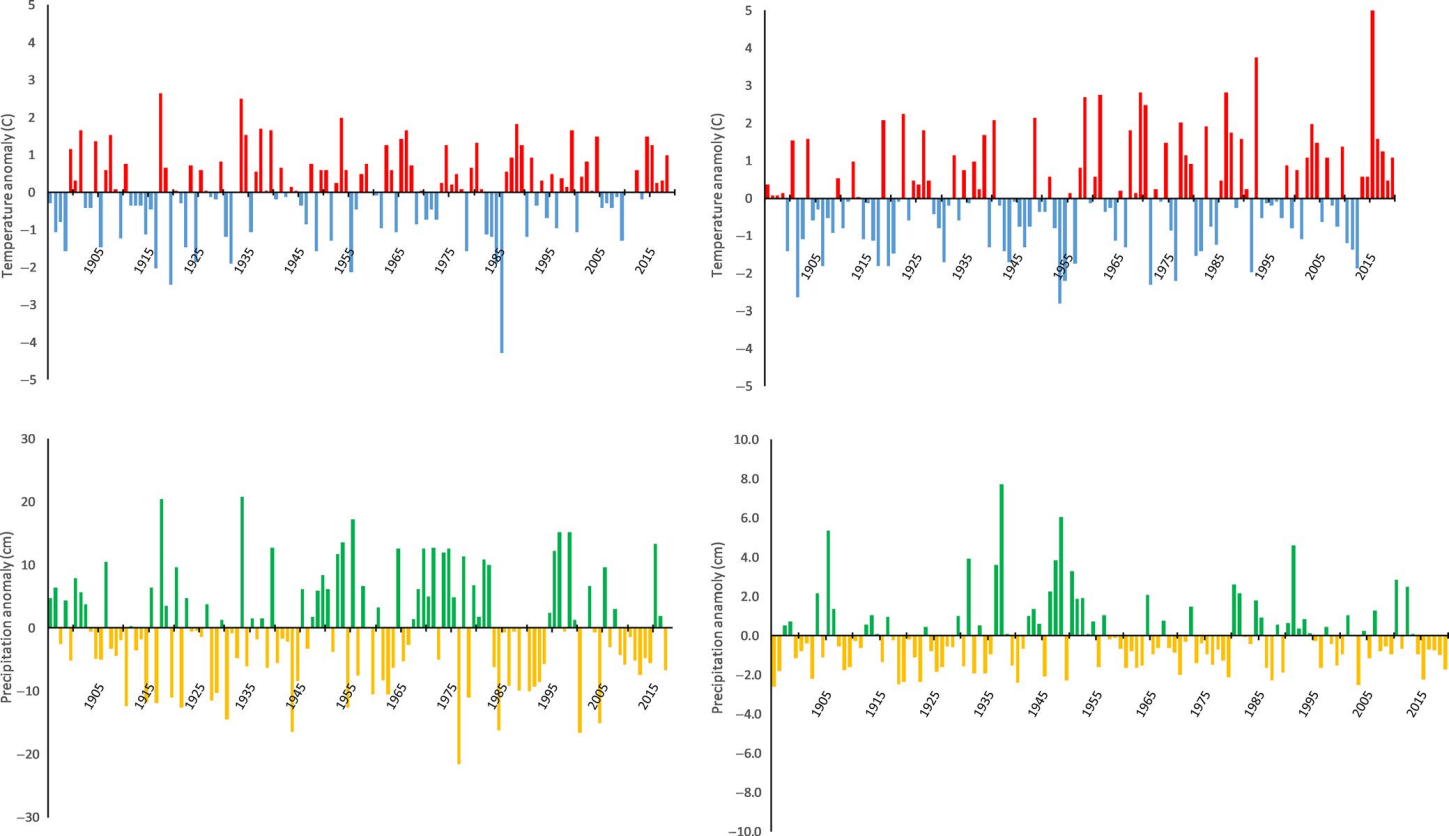
Temperature anomalies (°C) for December–February (upper left) and June (upper right), and precipitation anomalies (cm) for December–February (lower left) and June (lower right) for Yakima County, Washington. Anomalies were computing from the mean temperature, 1901 to 2000. Note that the y‐axes differ for the precipitation plots because most precipitation in our study area falls in winter (see Table 2)

It is important to note that our study area has experienced a consistent warming and drying trend over the last century that has been especially noticeable more recently (e.g., 2000–2019), with more frequent high temperature anomalies and less frequent low temperature anomalies (NOAA, 2019; Figure 2). For example, data from NOAA (2019) for our study area (Yakima County, Washington) over the seven years of our study showed that five of the years had experienced anomalously warm June and December–February temperatures (Figure 2). June of 2015 was particularly warm, with a mean temperature 5.2°C higher than average and daytime high temperatures regularly >32°C (NOAA, 2019). This was the warmest on record for the 124‐year period for which data are available (NOAA, 2019). On average, our study area also has experienced more frequent dry anomalies in June and December–February, with the exception of the winter of 2017 and June 2010 and 2012 (Figure 2).

### 2.6 Statistical analysis

Prior to conducting formal statistical analyses, we explored our stable isotope data to ensure that it met some basic assumptions. First, we examined δ^15^N and δ^13^C for normality using standard diagnostic plots, and they suggested that normality was a reasonable assumption for these data. We looked for differences in isotope niche breadth and dispersion by sex using Bayesian standard isotopic ellipses and found that 95% credibility intervals overlapped, suggesting no differences by sex. We also looked for differences in δ^15^N and δ^13^C by sex, treating nest as a repeated effect for nestlings, and found no significant differences. Therefore, we lumped samples from males and females in subsequent analyses.

### 2.7 Analysis—objective 1 (Factors affecting δ^15^N and δ^13^C)

We used an information–theoretic approach (Burnham & Anderson, 2002) to assess the influence of climate and habitat characteristics on δ^15^N and δ^13^C. We used δ^15^N and δ^13^C as response variables and compared support for a set of a priori models with different combinations of climate and habitat explanatory variables. We were interested in factors associated with higher δ^15^N, which is indicative of foraging at higher trophic levels (Pagani‐Núñez, Renom, Mateos‐Gonzalez, Cotín, & Senar, 2017; Tillberg, McCarthy, Dolezal, & Suarez, 2006; Vanderklift & Ponsard, 2003). We generated a separate set of models to explore factors affecting δ^13^C, which can vary for individuals foraging in arid versus mesic habitats and forested versus more open habitats (Chamberlain et al., 2000; Pagani‐Núñez et al., 2019).

For explanatory variables, we considered four PRISM covariates (climate covariates), six GNN covariates (habitat covariates), and elevation. As described above, climate covariates included those that were likely to affect arthropod abundance during the nestling period and included winter temperature and precipitation (winter_meant and winter_ppt), which affects snowpack and runoff, and June temperature, and precipitation (June_meant and June_ppt). We included habitat covariates that were hypothesized to affect White‐headed Woodpecker foraging in past research. We considered three covariates describing stand age and tree size because Dixon (1995) hypothesized that woodpeckers preferentially foraged on large trees in old‐growth forests: quadratic mean diameter of conifers (qmdc_dom; LEMMA, 2012), basal area weighted mean diameter of all live trees (mndbhba; LEMMA, 2012), and stand age (age_dom; LEMMA, 2012). We included two covariates describing basal area of tree species used for foraging by White‐headed Woodpeckers in central Washington in past studies (Lorenz et al., 2016): ponderosa pine basal area (pipo_ba; LEMMA, 2012) and fir basal area (abgr_psme_ba; grand fir and Douglas‐fir basal area combined; LEMMA, 2012). Last, we also included canopy cover of conifers (cancov_con; LEMMA, 2012) because Hollenbeck, Saab, and Frenzel (2011) suggested that areas with high canopy cover near nests provided important food resources for this species. We substituted nest elevation as a proxy for these habitat covariates in two models for the reasons mentioned above, in the section labeled *Habitat data*.

Prior to model building, we tested for correlations between all pairwise combinations of covariates and omitted variables with correlations >0.60 (Dormann et al., 2013). As a result, we excluded three potential covariates from our nestling models: age_dom, cancov_con, mndbhba. With the remaining eight covariates (Table 1), we developed a set of 11 nestling models to test hypotheses about factors affecting nestling White‐headed Woodpecker diet (Appendix 1). We included a set of three models that described climate effects, four models that described habitat effects, and three “combination models” which included both climate and habitat covariates. We also included a null model, which assumes that none of the models we considered were influential in woodpecker diet. The ecological hypotheses for these models, as well as a full list of covariates for each model, are provided in Appendix 1. We considered fewer than 20 potential models based on recommendations by Johnson and Omland (2004), who cautioned that large numbers of models can result in high ranking of models that are based on a spurious set of relationships rather than ecological hypotheses.

We used linear mixed models in SAS PROC GLIMMIX (SAS Institute, Inc., Cary, NC) to model the effects of our covariates on δ^15^N and δ^13^C. We included a repeated effect for each individual nest to account for within‐nest covariation caused by sampling multiple nestlings in each nest. We used a compound symmetry structure because we assumed that the pattern of nutrient distribution would be the same for all nestlings in a nest, and we had no reason to suspect a more complex relationship among nestling responses. This does not require the assumption that all nestlings in a nest are the same size or in the same nutritional state, only that the frequency and quality of feedings is the same for all nestlings. This was our expectation based on banding 131 nestlings and observing no consistent differences in body condition or size among individuals. We included a random effect for watershed. We used maximum likelihood estimation for computing AIC_c_ and restricted maximum likelihood for estimating model parameters for our best‐supported models. We looked for violations of model assumptions using student residual and standard diagnostic plots and assessed model fit by examining correlations between actual and predicted values for the best‐supported models.

We considered models in which Δ*i* < 2 relative to the top model to have the most support relative to other models that we considered. We present parameter estimates and 95% confidence intervals (CI) for the top‐ranked models. When 95% CI did not include 0, we concluded that the associated parameter had a strong effect on δ^15^N and δ^13^C given the other parameters in the model. We looked for violations of model assumptions using standard diagnostic plots and assessed model fit by examining correlations between actual and predicted values for the best‐supported models.

### 2.8 Analysis—objective 2 (δ^15^N and nest productivity)

We assessed whether δ^15^N varied in relation to nest productivity; that is, whether nests where nestlings were fed δ^15^N enriched diets produced more offspring. We used a repeated measures design in the lme4 package (Bates, Maechler, Bolker, & Walker, 2015) in R Studio version 1.2.5019 (R Core Development Team, R Foundation for Statistical Computing, Vienna, Austria). We treated nest productivity (number of fledglings) as an ordered variable and individual nestlings within nests as repeated factors.

### 2.9 Analysis—objective 3 (Niche width and overlap)

We compared isotopic niche width for nestlings by watershed and year. We plotted our isotope data for these groups in δ^13^C–δ^15^N space and estimated maximum likelihood and Bayesian standard ellipses using the SIBER package (Jackson et al., 2011) in R Studio. We estimated the size of each niche using Bayesian standard ellipse areas (SEA) and sample size‐corrected standard ellipse areas (SEA_c_). Total size of the SEA indicated trophic diversity within years or watersheds and the extent of overlap of ellipse areas indicated similarity in diet among years or watersheds. We also computed convex hull areas as a quick visual indication of the isotopic niche space occupied by each group. However, convex hulls are sensitive to sample size, and therefore, we considered SEA_c_ as a more accurate way to compare niche width among groups (Jackson et al., 2011).

## 3 RESULTS

**Figure 3:**
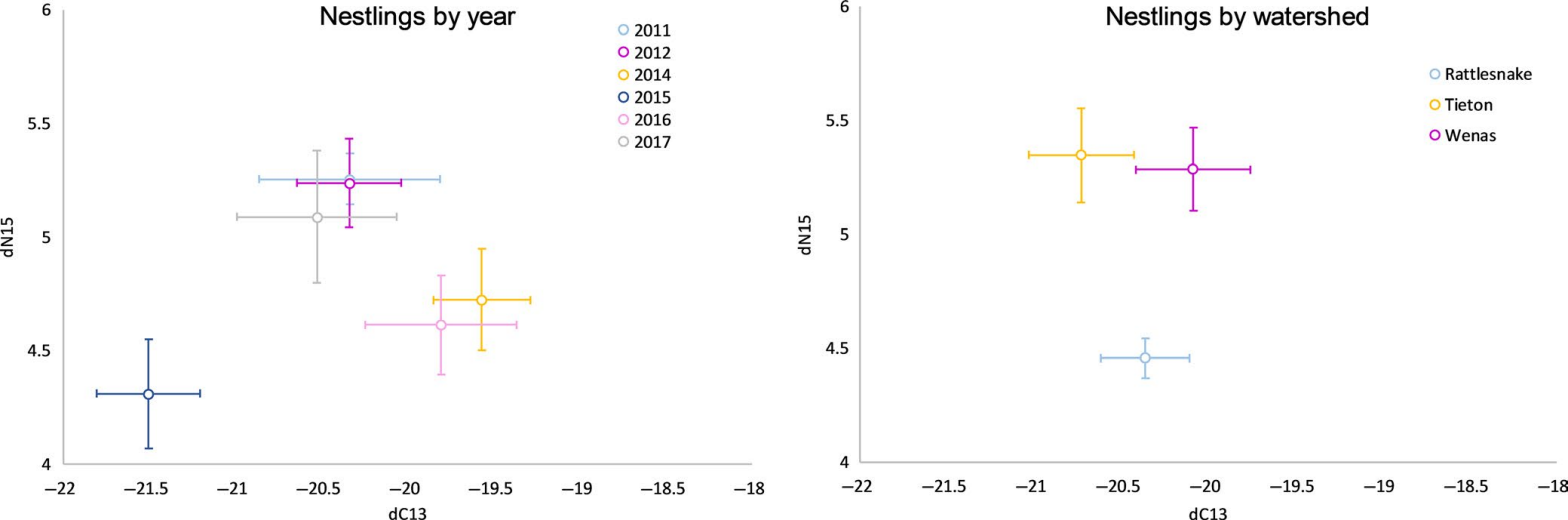
Plots showing mean and standard error of stable isotope ratios (δ^15^N vs. δ^13^C) for nestling White‐headed Woodpeckers by watershed (right) and year (left) in central Washington, from 2011 to 2017. We first averaged δ^15^N and δ^13^C within nests because nestlings within nests may not be independent. Nestling sample sizes refer to numbers of nests sampled; sample size in parentheses refers to the number of individual nestlings sampled. Nestling ratios indicate diet in June of year sampled

We collected feathers from 152 nestling woodpeckers at 64 nest sites. We found that δ^15^N was lowest in 2015 and for the Rattlesnake watershed (Figure 3). Mean basal area of ponderosa pine ranged between 5.1 m^2^/ha for the Wenas watershed and 10.6 m^2^/ha for the Tieton watershed. Mean basal area of firs varied between 2.4 m^2^/ha for the Wenas watershed and 11.4 m^2^/ha for the Rattlesnake watershed. Overall, forests in the Wenas watershed were more open (i.e., lower basal area) and were dominated by ponderosa pine. The other watersheds were closer to the Cascade Crest and had higher basal area of both firs and pines. For example, the Rattlesnake and Tieton watersheds contained about twice the basal area of firs and ponderosa pine compared to the Wenas (Table 2).

**Table 2:** Mean values (*SD*) at nest sites for covariates used in modeling factors affecting variation in δ^13^C and δ^15^N stable isotopes for 152 nestling White‐headed Woodpeckers in central Washington, by watershed

	Rattlesnake Watershed (*n* = 51)	Tieton Watershed (*n* = 30)	Wenas Watershed (*n* = 14)
*Habitat factors*			
Abgr_psme_ba (m^2^/ha)	11.4 (3.0)	10.0 (2.9)	2.4 (0.9)
Nest_elevation (m)	1,059.6 (133.6)	1,010.9 (133.5)	820.6 (58.8)
Pipo_ba (m^2^/ha)	7.1 (3.0)	10.6 (1.6)	5.1 (1.7)
Qmdc_dom (cm)	29.3 (3.8)	29.7 (2.3)	19.9 (2.9)
*Climate factors*			
June_meant (C)	13.3 (2.0)	12.3 (2.6)	12.0 (0.7)
June_ppt (mm)	18.2 (9.7)	21.0 (11.9)	27.8 (12.5)
Winter_meant (C)	−1.0 (1.7)	−1.1 (1.6)	−1.0 (0.3)
Winter_ppt (mm)	381.2 (133.7)	390.5 (152.2)	242.9 (53.4)

Note

Only watersheds with at least 10 nests are included in this table. Sample sizes indicate number of independent nests where feather samples were collected.

Based on data obtained from PRISM, mean temperature ranged between 12.0 to 14.0°C in June and −1.0 and −1.3°C in winter among watersheds (Table 2). Precipitation varied by longitude and elevation. The Tieton and Rattlesnake watersheds were higher in elevation and farther west (closer to the Cascade Crest; Figure 1) and as expected had higher amounts of winter precipitation (Table 2).

The top‐ranked model for nestling δ15N included june_meant, winter_meant, winter_ppt, and nest_elevation_m. (Table 3). In this model, most of the effect was assigned to winter_meant and this parameter estimate was significant and inversely related to δ^15^N in nestlings (Table 4). Colder winter temperatures were associated with an increase in the δ^15^N of nestlings. None of the models that included habitat factors ranked high in our analysis. For δ^13^C, none of the covariates had a measurable effect. The top three models with Δ*_i_* < 2 included our null model, a model with only June_ppt, and a model with only qmdc_dom (Table 5). None of the estimates in these models were significant; all had 95% CI that overlapped zero (Table 4).

**Table 3:** Support for models examining climate and habitat on δ^15^N ratios for 152 nestling White‐headed Woodpeckers in central Washington, 2011–2017

Model number	Model class	Covariates	AIC_c_	*k*	Δ*_i_*	*w_i_*
10	Climate and habitat (Global without GNN)	june_meant, winter_meant, winter_ppt, nest_elevation_m	118.41	5	0.00	0.593
3	Climate	june_meant, winter_meant, winter_ppt	120.54	4	2.13	0.205
2	Climate	winter_meant, winter_ppt	121.91	3	3.50	0.103
9	Climate and habitat (Global with GNN)	june_meant, winter_meant, winter_ppt, pipo_ba, abgr_psme_ba, qmdc_dom	123.81	7	5.40	0.040
8	Climate and habitat	june_meant, pipo_ba, abgr_psme_ba	124.49	4	6.08	0.028
1	Climate	june_meant	124.55	2	6.14	0.028
5	Habitat	nest_elevation_m	130.70	2	12.29	0.001
6	Habitat	qmdc_dom	130.84	2	12.43	0.001
7	Habitat	pipo_ba, abgr_psme_ba	132.87	3	14.46	0.000
4	Habitat	pipo_ba, abgr_psme_ba, qmdc_dom	133.52	4	15.11	0.000
11	Null model	None	134.32	1	15.91	0.000

Note

Covariates are described in Table 1.

**Table 4:** Covariate estimates and 95% confidence intervals (CI) for top‐ranked models (<2 Δ*_i_*) explaining variation in δ^15^N and δ^13^C ratios in feathers of 152 nestling White‐headed Woodpeckers in central Washington, 2011–2017

Covariates	Estimate	*SE*	*t* value	*p*	Lower 95% CI	Upper 95% CI
Top‐ranked δ^15^N model						
winter_meant	−0.1315	0.0482	−2.7300	.0085	−0.2281	−0.0350
nest_elevation_m	−0.0013	0.0006	−1.9500	.0556	−0.0026	0.0000
june_meant	−0.0602	0.0398	−1.5200	.1351	−0.1398	0.0193
winter_ppt	0.0003	0.0006	0.5500	.5875	−0.0009	0.0016
Top‐ranked δ^13^C models						
Model 1						
june_ppt	0.0141	0.0142	0.9900	.3239	−0.0142	0.0423
Model 6						
qmdc_dom	−0.0227	0.0373	−0.6100	.5443	−0.0973	0.0518

Note

Estimates considered significant if confidence intervals did not include 0.

**Table 5:** Support for models examining climate and habitat on δ^13^C ratios for 152 nestling White‐headed Woodpeckers in central Washington, 2011–2017

Model number	Model class	Covariates	AIC_c_	*k*	Δ*_i_*	*w_i_*
11	Null model	None	402.41	1	0.00	0.325
1	Climate	june_ppt	403.50	2	1.09	0.188
6	Habitat	qmdc_dom	404.13	2	1.72	0.137
5	Habitat	nest_elevation_m	404.51	2	2.10	0.114
2	Climate	winter_meant, winter_ppt	405.02	3	2.61	0.088
7	Habitat	pipo_ba, abgr_psme_ba	405.65	3	3.24	0.064
3	Climate	june_ppt, winter_meant, winter_ppt	406.67	4	4.26	0.039
4	Habitat	pipo_ba, abgr_psme_ba, qmdc_dom	407.80	4	5.39	0.022
10	Climate and habitat (Global without GNN)	june_ppt, winter_meant, winter_ppt, nest_elevation_m	408.72	5	6.31	0.014
9	Climate and habitat (Global with GNN)	june_ppt, winter_meant, winter_ppt, pipo_ba, abgr_psme_ba, qmdc_dom	410.27	7	7.86	0.006
8	Climate and habitat	june_ppt, pipo_ba, abgr_psme_ba	411.58	4	9.17	0.003

We found a marginally nonsignificant effect of δ^15^N on nest productivity (*F*_53,3_ = 2.40, *p* = .0783). If the null hypothesis was true (δ^15^N does not affect nest productivity), then there was about a 1 in 12 chance that a random sample would yield a difference of this magnitude. Overall, there was a slight trend for δ^15^N to increase 0.06 for every increase in one nestling fledged from each nest.

**Figure 4:**
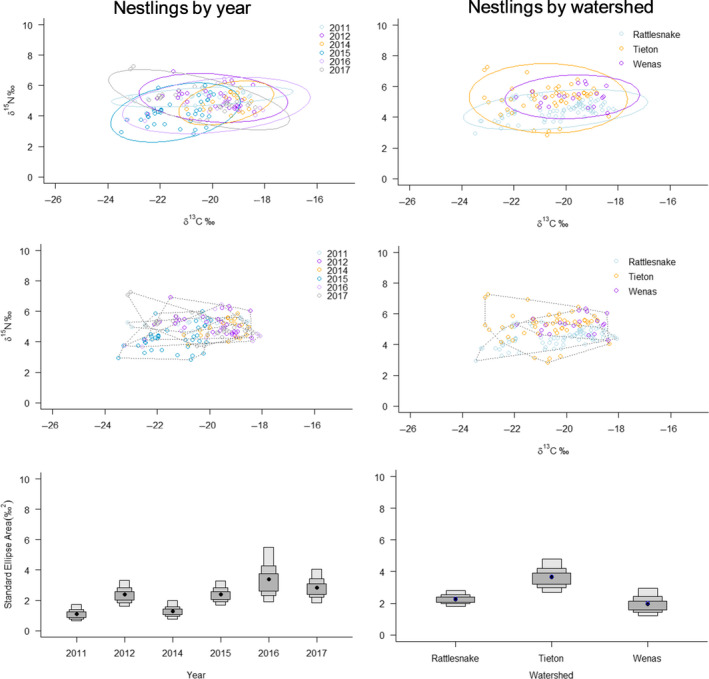
Maximum likelihood standard ellipses (top), convex hulls (middle), and Bayesian standard ellipse area credibility intervals (bottom; 50%, 75%, and 95%) depicting δ^15^N vs. δ^13^C stable isotope niche widths for 152 nestling White‐headed Woodpeckers sampled in central Washington, from 2011 to 2017. Plots show comparisons by year (left) and by watershed (right). Watersheds are described in Figure 1 and Table 2

Stable isotope niche width was lowest in 2011 and highest in 2016 (Table 6, Figure 4). Among watersheds, niche width was lowest in Wenas, a site with low tree species diversity compared to the other two watersheds (Table 2). Stable isotope niches overlapped 62% on average among years. The smallest degree of overlap occurred for the SEA of 2011, which overlapped the SEAs of 2015 to 2017 by only 30%–31% (Table 7).

**Table 6:** Stable Isotope niche width for 152 nestling White‐headed Woodpeckers in central Washington by year and watershed

Nestlings by year	2011	2012	2014	2015	2016	2017
TA (Convex Hull Total Area)	2.7	8.4	2.9	8.3	6.8	7.4
SEA (Standard Ellipse Area)	1.1	2.3	1.3	2.4	3.3	2.7
SEAc (Standard Ellipse Area corrected for small sample sizes)	1.1	2.4	1.3	2.4	3.6	2.8

Nestlings by watershed	Rattlesnake	Tieton	Wenas			
TA (Convex Hull Total Area)	7.8	14.1	4.8			
SEA (Standard Ellipse Area)	2.2	3.6	1.9			
SEAc (Standard Ellipse Area corrected for small sample sizes)	2.3	3.7	2.0			

**Table 7:** Overlap between fitted ellipses for 152 nestling White‐headed Woodpeckers occupying three watersheds in central Washington, 2011–2017

Nestlings by year	2011	2012	2014	2015	2016	2017
2011	–	0.40	0.45	0.31	0.30	0.30
2012	0.84	–	0.95	0.52	0.60	0.71
2014	0.52	0.55	–	0.33	0.37	0.39
2015	0.67	0.52	0.61	–	0.57	0.47
2016	0.93	0.88	1.00	0.84	–	0.72
2017	0.76	0.83	0.83	0.56	0.57	–

Nestlings by watershed	Rattlesnake	Tieton	Wenas			
Rattlesnake	–	0.53	0.64			
Tieton	0.86	–	0.93			
Wenas	0.57	0.51	–			

Note

Data are the proportion of standard ellipse area (SEA) from year or watershed indicated in rows that is occupied SEA from year or watershed indicated in columns. For example, reading the second column, first row, 0.40 of the SEA of nestlings in 2012 was overlapped by 2011. See Figure 4 for a visualization of the amount of overlap for each of these categories.

## 4 DISCUSSION

We found that nestling White‐headed Woodpeckers were fed diets with low δ^15^N values following warm winters. Low winter precipitation, high June mean temperature, and high elevations were also associated with low δ^15^N values, though their effects were weaker than mean winter temperature. No habitat factors were influential in our top nestling models. The lack of strong habitat effects suggests this species was foraging at a similar trophic level irrespective of stand‐level attributes in our study (e.g., δ^15^N was similar among nestlings captured in different habitats). This may be partially due to habitat conditions that did not vary widely across our study area. In contrast, climate conditions varied considerably over the 6 years of our study, two of which (2015 and 2016) were among the warmest on record (Figure 3). The year 2015 was especially warm and in which nestlings had extremely low δ^15^N values. Thus, our ability to make inferences about the impact of habitat on diet may be limited, but we found a strong effect of winter temperature on δ^15^N.

Higher δ^15^N values are often associated with higher trophic level foraging, which is sometimes considered beneficial and linked to higher survival (Hodum & Hobson, 2000; St. John Glew et al., 2019). However, there are no formal tests of the impact of δ^15^N on White‐headed Woodpecker nestling survival and development. We tested for and found a marginally nonsignificant relationship between δ^15^N and nest productivity; nest productivity declined with δ^15^N, suggesting that climate may also impact reproductive output.

For the most robust conclusions, future studies are needed that measure isotopic signatures of arthropod prey to directly link variation in isotopic signatures with food items. Lacking this information, we can only speculate on the possible dietary items that resulted in these effects using other research studies as a guide. Kozma and Kroll (2013) conducted a study of White‐headed Woodpecker nestling provisioning in our study area. They found that the most common arthropod groups fed to nestlings were wood‐boring beetle larvae (25%), caterpillars (23%), and ants (18%). In other research, ants have reportedly high δ^15^N compared to caterpillars and wood‐boring beetle larvae (Bennett & Hobson, 2009) because ants are predators on other arthropods and thus feed at a higher trophic level (Feldhaar, Gebauer, & Blüthgen, 2010). Considered together, this suggests that adult White‐headed Woodpeckers may feed nestlings a diet with more insect larvae low in δ^15^N (such as wood‐boring beetle grubs and caterpillars) following warm winters, whereas they feed nestlings a diet with more ants (e.g., higher in δ^15^N) following colder winters. Because of their higher trophic level position, ants may provide a more significant source of protein (Noyce, Kannowski, & Riggs, 1997). In contrast, caterpillars, and presumably other insect larvae such as those of wood‐boring beetles, contain high levels of fat and water (Brodmann & Reyer, 1999; Kouřaminská & Adámková, 2016). In addition, insect larvae contain less chitin than adult insects and may be easier to digest (Brodmann & Reyer, 1999; Kouřaminská & Adámková, 2016; Orłowski, Frankiewicz, & Karg, 2017). Adult woodpeckers may face trade‐offs when feeding young. Prey that are rich in calories and water are lower in nitrogen (insect larvae), while nitrogen rich prey are low in calories and high in indigestible chiton (ants). Another consideration, however, is that if prey availability varies with climate, adults may have relatively little control over the types of food they feed to nestlings. Future studies that explore the effects of different prey groups on nestling development would be valuable, as would studies of prey availability in relation to weather and climate in this region. Such studies would shed light on how changes in climate impact the abundance of specific arthropod groups, woodpecker diet, and nestling condition.

Higher δ^15^N ratios following cold winters may be associated with poor survival of caterpillars or beetle larvae, which leads woodpeckers to forage more on ants. For example, frequent exposure to cold temperatures reduces the long‐term survival in spruce budworm (*Choristoneura occidentalis*; Marshall & Sinclair, 2015), the larvae of which is a common food source for White‐headed Woodpeckers (Lorenz et al., 2016). However, many factors can influence annual survival of such insects (Gray, 2013) and more study is needed before causal links between temperature and individual dietary items in woodpeckers can be elucidated. Our study indicates that significant changes in nestling diet were linked with winter temperatures and that under future climate scenarios, White‐headed Woodpecker nestling diet may shift in favor of lower trophic level prey. The impact of such changes on woodpecker populations merits further study.

We observed considerable niche overlap, although some patterns did emerge. Isotopic niche width was lowest in watersheds dominated by ponderosa pine and wider (indicating a greater variety of prey items) in watersheds with both pines and firs. This is consistent with our expectation that diversity of prey should be higher in stands composed of three tree species, than those composed of a single tree species. In our study area, firs occur in areas where winter snowpack is greater and lingers longer into spring, whereas ponderosa pine is dominant on more arid sites. Thus, diversification in the White‐headed Woodpecker diet appears to be associated with greater tree species diversity. Woodpeckers may benefit from sites containing a wide variety of prey because such sites provide a buffer against shortages in one or two food sources. However, there are likely some trade‐offs. For example, sites with prolonged snowpack often are associated with delayed plant and arthropod phenology, which may delay woodpecker breeding.

While our study provides insights into White‐headed Woodpecker foraging ecology, there are limitations that bear consideration. First, as noted above, stable isotope ratios do not provide information on the specific diet items consumed by woodpeckers. Without corresponding stable isotope ratios from arthropods in our study area and information on their availability, we do not know for certain what prey woodpeckers were consuming. Thus, our speculations about ants forming a greater part of the nestling diet following cool winters need to be verified with isotopic data. Additionally, we only sampled nestlings in our study and adult diets may be influenced by other factors. Last, we are limited in our ability to make conclusions about woodpecker diet during cool or wet years because every year of our study was anomalously warm and dry.

## 5 CONCLUSION

We found that warm winter weather was associated with lower trophic level diets (lower δ^15^N) in nestling White‐headed Woodpeckers. Future studies should explore whether this has consequences for nestling development and long‐term survival. In our study, we were able to test for differences in nest productivity by δ^15^N. We found a marginally nonsignificant effect, and lower δ^15^N was associated with lower nest productivity (number of young fledged). We did not find strong effects of habitat factors on isotopic ratios, although habitat features at our sites may have been too similar for us to detect habitat effects. We did find that sites with a more diverse tree community were associated with a more diverse diet. This suggests that at least within the limits imposed by their habitat preferences, this woodpecker will utilize a range of tree species for foraging, when available. Such adaptability will be important if this species is to persist in the rapidly changing climate of western North America.

## Conflict of interest

None declared.

## AUTHOR CONTRIBUTION

**Teresa J. Lorenz:** Conceptualization (lead); Data curation (lead); Formal analysis (supporting); Funding acquisition (lead); Investigation (equal); Methodology (lead); Project administration (lead); Resources (lead); Software (supporting); Supervision (lead); Validation (equal); Visualization (equal); Writing‐original draft (lead); Writing‐review & editing (equal). **Jeffrey M. Kozma:** Conceptualization (supporting); Data curation (supporting); Investigation (equal); Methodology (supporting); Resources (supporting); Software (supporting); Validation (equal); Visualization (equal); Writing‐original draft (supporting); Writing‐review & editing (equal). **Patrick G. Cunningham:** Conceptualization (supporting); Data curation (supporting); Formal analysis (lead); Investigation (equal); Methodology (supporting); Software (supporting); Validation (equal); Visualization (equal); Writing‐original draft (supporting); Writing‐review & editing (equal).

## Data Availability

Raw data are available at: https://doi.org/10.6084/m9.figshare.12088977

## Appendix 1

Final model hypotheses and covariates list for effects of climate and habitat on nestling white‐headed woodpecker δ^15^N and δ^13^C values. Variables are defined in Table 1. Climate variables were obtained from PRISM (PRISM Climate Group, 2019: https://prism.oregonstate.edu/). Habitat variables were obtained from GNN data from LEMMA (2012: https://lemma.forestry.oregonstate.edu/).

### Climate models

1. Model hypothesis 1: Nestling diet is influenced predominately by weather during the time contour feathers are developing in June. June_meant and June_ppt were correlated in our exploratory analysis. We included June_meant in our models for δ^15^N because we expected temperature to more directly impact plant phenology and thus arthropod abundance relative to δ^15^N assimilation. However, for δ^13^C we included June_ppt because of past research suggesting that rainfall affects δ^13^C in other bird species. Variables include: June_meant (δ^15^N models) and June_ppt (δ^13^C models)
2. Model hypothesis 2: Nestling diet is influenced predominately by climate and weather events in the preceding winter, especially snowpack and extreme cold events. Note that mean winter temperature is highly correlated with winter minimums. Variables include: winter_meant and winter_ppt
3. Model hypothesis 3: Nestling diet is influenced by weather conditions in winter, when most precipitation falls on our study area, and in June when nestlings are growing contour feathers. Variables include: June_meant (δ^15^N models), June_ppt (δ^13^C models), winter_meant, and winter_ppt

### Habitat models

1. Model hypothesis 4: Nestling diet is influenced predominately by habitat factors; climate does not play a role in diet variation. Variables include: abgr_psme_ba, pipo_ba, and qmdc_dom
2. Model hypothesis 5: Nestling diet is influenced predominately by habitat, but the GNN covariates are too inaccurate (or too coarse in scale) to use in modeling for this particular ecological question. Therefore, we used nest elevation as a proxy for the GNN habitat variables. Variables include: nest_elevation_m
3. Model hypothesis 6: The presence of large trees alone predicts nestling diet, based on the premise of Dixon (1995) that this species benefits from old‐growth forests for better foraging conditions. Variables include: qmdc_dom
4. Model hypothesis 7: Nestling diet is influenced predominately by forest composition, but not tree size. Variables include: abgr_psme_ba and pipo_ba

### Combination (climate and habitat) models

1. Model hypothesis 8: Nestling diet is influenced predominately by forest composition and weather conditions when feathers are growing in June. Variables include: abgr_psme_ba, pipo_ba, June_meant (δ^15^N models), and June_ppt (δ^13^C models)
2. Model hypothesis 9: Nestling diet is influenced by all the variables we considered (almost the global model, but with nest_elevation_m excluded). Variables include: abgr_psme_ba, June_meant (δ^15^N models), June_ppt (δ^13^C models), pipo_ba, qmdc_dom, winter_meant, and winter_ppt
3. Model hypothesis 10: Nestling diet is influenced by all the variables we considered (almost the global model) but the GNN variables are too inaccurate to use in modeling. Therefore, we used nest elevation as a proxy for the GNN habitat variables. Variables include: June_meant (δ^15^N models), June_ppt (δ^13^C models), nest_elevation_m, winter_meant, and winter_ppt

### Null model

1. Model hypothesis 11: Nestling diet is influenced by factors we did not consider. Variables include: none (null model)
